# Influence of Clinical Status and Parasite Load on Erythropoiesis and Leucopoiesis in Dogs Naturally Infected with *Leishmania (Leishmania) chagasi*

**DOI:** 10.1371/journal.pone.0018873

**Published:** 2011-05-10

**Authors:** Raquel Trópia de Abreu, Maria das Graças Carvalho, Cláudia Martins Carneiro, Rodolfo Cordeiro Giunchetti, Andréa Teixeira-Carvalho, Olindo Assis Martins-Filho, Wendel Coura-Vital, Rodrigo Corrêa-Oliveira, Alexandre Barbosa Reis

**Affiliations:** 1 Laboratório de Imunopatologia, Núcleo de Pesquisas em Ciências Biológicas, Departamento de Análises Clínicas, Universidade Federal de Ouro Preto, Ouro Preto, Minas Gerais, Brazil; 2 Laboratório de Hematologia Clínica, Faculdade de Farmácia, Universidade Federal de Minas Gerais, Belo Horizonte, Minas Gerais, Brazil; 3 Laboratório de Imunopatologia, Núcleo de Pesquisas em Ciências Biológicas, Departamento de Ciências Biológicas, Universidade Federal de Ouro Preto, Ouro Preto, Minas Gerais, Brazil; 4 Laboratório de Biomarcadores de Diagnóstico e Monitoração, Centro de Pesquisas René Rachou, Fundação Oswaldo Cruz, Belo Horizonte, Minas Gerais, Brazil; 5 Laboratório de Imunologia Celular e Molecular, Centro de Pesquisas René Rachou, Fundação Oswaldo Cruz, Belo Horizonte, Minas Gerais, Brazil; Centers for Disease Control and Prevention, United States of America

## Abstract

**Background:**

The bone marrow is considered to be an important storage of parasites in *Leishmania*-infected dogs, although little is known about cellular genesis in this organ during canine visceral leishmaniasis (CVL).

**Methodology/Principal Findings:**

The aim of the present study was to evaluate changes in erythropoiesis and leucopoiesis in bone marrow aspirates from dogs naturally infected with *Leishmania chagasi* and presenting different clinical statuses and bone marrow parasite densities. The evolution of CVL from asymptomatic to symptomatic status was accompanied by increasing parasite density in the bone marrow. The impact of bone marrow parasite density on cellularity was similar in dogs at different clinical stages, with animals in the high parasite density group. Erythroid and eosinophilic hypoplasia, proliferation of neutrophilic precursor cells and significant increases in lymphocytes and plasma cell numbers were the major alterations observed. Differential bone marrow cell counts revealed increases in the myeloid:erythroid ratio associated to increased numbers of granulopoietic cells in the different clinical groups compared with non-infected dogs.

**Conclusions:**

Analysis of the data obtained indicated that the assessment of bone marrow constitutes an additional and useful tool by which to elaborate a prognosis for CVL.

## Introduction

Visceral leishmaniasis (VL), which is caused by *Leishmania* (*Leishmania*) *infantum* [syn. *Leishmania* (*Leishmania*) *chagasi*], is endemic in over 88 countries within Europe and Latin America, and is transmitted by the bite of the female sand fly (phlebotomine) [1]. The vast majority (*ca.* 90%) of VL cases worldwide occur in Bangladesh, Brazil, India and Sudan. In Brazil, for example, some 37,294 new human cases of VL have been reported in the last 16 years [2]. Canine visceral leishmaniasis (CVL) cause a great impact in Brazilian public health, because, domestic dogs are the most important VL peridomicile reservoirs in urban and periurban areas, since both asymptomatic and symptomatic dogs are equally infectious to the vectors [3]. Moreover, based on either the similarity of clinical signs observed in human and dogs and the evolution of natural history of the disease, CVL has been suggested as a good model to better understand the pathogenesis of the human disease [4], [5].

A number of studies relating to CVL have concerned the biochemical-haematological alterations in dogs that had been either naturally or experimentally infected with *Leishmania*. The findings include normocytic/normochromic anaemia, leucopoenia, thrombocytopenia, an increase in total serum proteins with hyperglobulineamia and hypoalbumineamia, decreased albumin/globulin ratio [5], [6], [7] and alterations in the hepatic enzymes aspartate aminotransferase and alanine aminotransferase [8].

Recent investigations have revealed various correlations between parasite density or clinical status and alterations in immunological, biochemical-haematological and histopathological biomarkers in different lymphoid tissues [5], [7], [9], [10].The correlation of major peripheral blood phenotypic markers with clinical status and tissue parasite densities reported by our group [11], [12] highlights the complexity of the cellular immunological events related to the natural infection of dogs with *Leishmania chagasi*. We demonstrated that lower frequencies of circulating B cells and monocytes are important markers of severe CVL, whereas an increased level of CD8^+^ lymphocytes appears to be the major phenotypic feature of the asymptomatic infection.

Evaluation of humoral immune response during CVL revealed that asymptomatic dogs and animals presenting low parasite density were associated with an increase in IgG1, while symptomatic dogs and those presenting high parasite density were associated with increases of IgG, IgG2, IgM, IgA and IgE immunoglobulins [7], [10].

Such pioneer findings indicated a correlation between clinical status of CVL and tissue parasite density. In this context, we have shown that asymptomatic dogs exhibit low parasite density while symptomatic dogs present high parasite density in various tissues including skin, bone marrow, spleen, liver and lymph node [5], [7], [9], [11], [13], [14]. The skin and spleen are the major sites of high parasite density during ongoing CVL considering clinical status, [5], [12] although parasite densities in the bone marrow and spleen provide the most reliable parasitological markers to decode the clinical status of CVL [5].

Bone marrow is a major haematopoietic organ and a primary lymphoid tissue, besides it's considered to be an important storage of parasites in *Leishmania*-infected dogs. This infection could cause dysfunction in the production of erythrocytes, granulocytes, monocytes, lymphocytes and platelets. There is little information in the literature about bone marrow cellularity in dogs naturally infected with *L. chagasi,* although some reports are available concerning the pathological alterations in this organ during CVL. Thus, the occurrence of hyperplasia with increased neutrophils and granulocyte precursors gives rise to an increase in the myeloid: erythroid (M:E) ratio, whilst additional alterations, including increases in monocytes, macrophages, plasma cells and the number of Mott cells, indicate an antigenic stimulation associated with infection of the bone marrow compartment [15], [16]. Additionally, Foglia Manzillo *et al*. [17] related pathological alterations in the bone marrow with CVL in respect of three clinical groups, namely, asymptomatic, oligosymptomatic and symptomatic animals, and found major modifications involving the red cell series (erythrophagocytosis, erythroid hypoplasia and dysplasia) and the white cell series (myeloid, neutrophilic and eosinophilic hyperplasia).

It is clear, therefore, that clinical evolution of VL in naturally infected dogs promotes distinct alterations in bone marrow cells, whilst elevated bone marrow parasite density could provoke alterations in myelopoiesis. It is possible that such modifications in the bone marrow could result in and explain the changes in peripheral blood. The present study represents a detailed investigation of the alterations in leucopoiesis and erythropoiesis in the bone marrow of dogs naturally infected with *L. chagasi* and presenting different clinical status and distinct patterns of bone marrow parasite density.

## Materials and Methods

### Selection of dogs

Details of the study were submitted to and approved by the Ethical Committee for the use of Experimental Animals of the Universidade Federal de Ouro Preto, Brazil (Protocol number 2007/83). A total of 187 mixed-breed adult dogs (93 male and 94 female) aged between 2 and 6 years were captured by the Centro de Controle de Zoonoses, Belo Horizonte, Minas Gerais, Brazil. Animals were selected for inclusion in the study on the basis of the results of an immunofluorescence antibody test (IFAT), which is considered to be the “gold standard” immunological assay for the diagnosis of CVL [5]. Animals (*n* = 28, being 14 male and 14 female) with negative IFAT results at 1∶40 sera dilution and negative parasitological examinations for *Leishmania* were classified as non-infected dogs and included in the NID group. Dogs (*n* = 159) presenting IFAT titres ≥1∶40 were considered to be positive with respect to CVL, and these animals were included into one of the groups constituted of infected animals (asymptomatic, oligosymptomatic or symptomatic). In each case, infection with *L. chagasi* was confirmed serologically using enzyme-linked immunosorbent assay (ELISA)- extract (soluble antigens from *L. infantum* (syn. *chagasi*) promastigote-MHOM/BR/1972/BH46) and ELISA rK39, as described previously [5], and/or by parasitological examination as described below.

### Bone marrow aspiration and determination of bone marrow cellularity

Animals were sedated with an intravenous dose (8 mg/kg body weight) of sodium thiopental (Thionembutal®; Abbott Laboratories, São Paulo, Brazil), and bone marrow fluid was removed from the ventral region of the sternum or from the iliac crest. The bone marrow aspirates were used to obtain slide smears that were Giemsa-stained and examined under the optical microscope in order to identify amastigote forms of *Leishmania*. Leucopoietic and erythropoietic alterations were evaluated by differential counting of 500 cells with reference to the cellular classifications of Penny [18] and Jain [19]. In order to evaluate bone marrow responses in dogs naturally infected with *L. chagasi* and showing different clinical statuses and patterns of bone marrow parasite density, numerical assessments of bone marrow cellularities were made on the basis of the maturation index ratios defined by Hoff *et al*. [20] and Mischke *et al*. [21] (Table 1).

**Table 1:** Calculation of Maturation Index Ratios for the numerical determination of cellularity in the bone marrow.

Maturation Index Ratio	Definition
M:E	Number of myeloid cells/Number of nucleated erythroid cells
I:M	Number of myeloblasts + promyelocytes + myelocytes/Number of metamyelocytes + bands + segmented
I:Mg	Number of myeloblasts + promyelocytes + neutrophilic myelocytes/Number of neutrophilic metamyelocytes, bands and segmented neutrophils
MMI (1/I:Mg)	Number of neutrophilic metamyelocytes, bands and segmented neutrophils/Number of myeloblasts + promyelocytes + neutrophilic myelocytes
I:Me	Number of proerythroblasts + basophilic erythroblasts/Number of polychromatic and orthochromatic erythroblasts
EMI (1/I:Me)	Number of polychromatic and orthochromatic erythroblasts/Number of proerythroblasts + basophilic erythroblasts

M indicates myeloid; E, erythroid; I, immature; M, mature; I:Mg, immature: mature (granulopoiesis); MMI, myeloid maturation index; I:Me, immature: mature (erythropoiesis); EMI, erythroid maturation index.

### Clinical and parasitological evaluations

Mongrel dogs that were seropositive by IFAT were classified into three distinct groups on the basis of the presence or absence of clinical signs Mancianti *et al*. [22]. The categories were: “asymptomatic” (AD, *n* = 50, being 24 male and 26 female) relating to dogs with no signs indicative of the disease; “oligosymptomatic” (OD, *n* = 44, being 24 male and 20 female) relating to dogs presenting a maximum of three clinical signs including opaque bristles, localised alopecia, or moderate loss of weight; and “symptomatic” (SD, *n* = 65, being 32 male and 33 female) relating to dogs exhibiting the characteristic clinical signs of VL including opaque bristles, severe loss of weight, onycogryphosis, cutaneous lesions, apathy and keratoconjunctivitis.

Parasitological analysis and the determination of parasite density were carried out on bone marrow smears and the results expressed as “Leishman Donovan Units” (LDU index) corresponding to the number of *Leishmania* amastigotes per 1000 nucleated cells of the leucopoietic series [5], [11]. Bone marrow parasite density was initially classified as low (LP), medium (MP) and high (HP) according to bone marrow-specific LDU indices categorised statistically into tertiles as follows: LP (1–3), MP (4–14) and HP (15–660). This approach strengthened the statistical analysis by producing well-balanced numbers of dogs in each subgroup (LP = 60; MP = 51; HP = 48).

### Statistical analysis

Statistical analyses were performed using GraphPad Prism 4.03 (Prism Software, Irvine, CA, USA) and Minitab Statistical Software 13.20 (Minitab Inc., Pennsylvania, USA). Since the evaluated data were non-parametric, Kruskal-Wallis test was used for the comparative study between groups, followed by realization Dunn's test. Spearman's rank correlation (rs) was computed to investigate associations between parasite density and clinical status parameters. In all cases, differences were considered significant when the probabilities of equality (p-values) were ≤0.05.

## Results

### Dogs with low and high bone marrow parasite density and all clinical groups show hypoplasia of orthochromatic erythroblast population

**Figure 1:**
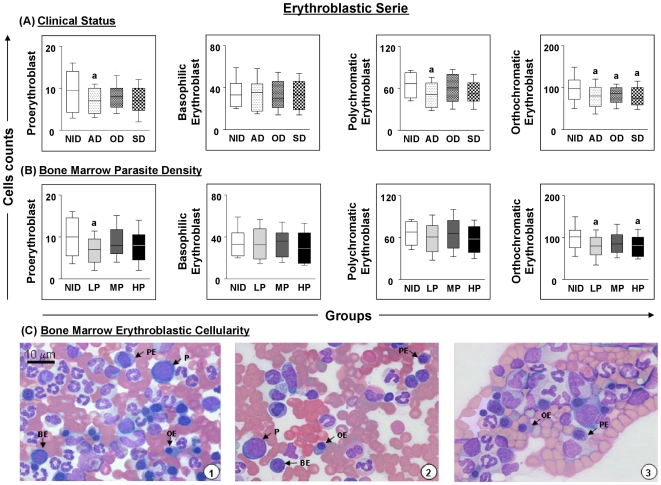
Profiles of the erythroblastic cell series in dogs naturally infected with *Leishmania chagasi*. Panel **A** - animals categorised according to their clinical status into asymptomatic (AD = white boxes with small points), oligosymptomatic (OD = white boxes with large points), symptomatic (SD = white boxes with squares) or non-infected (NID = white boxes) groups. Panel **B** - animals categorised according to bone marrow parasite density into low (LP = gray boxes), medium (MP = dark gray boxes) and high (HP = black boxes) parasite density and non-infected (NID = white boxes) groups. Absolute cell counts are presented in a box-plot format, the median, maximum and minimum values represents the interquartile range. Significant differences (p<0.05) with respect to the NID group are indicated by the letter ‘a’. Panel **C** - cellularity of the erythroblastic series observed in the bone marrow of dogs assessed in this study. Slides were stained with Giemsa; bar  = 10 µm. P indicates proerythroblast; BE, basophilic erythroblast; PE, polychromatic erythroblast; OE, orthochromatic erythroblast.

Alterations in the red cells present in animals within different clinical groups and with different parasite densities tended towards decreasing numbers of proerythroblasts, and polychromatic and orthochromatic erythroblasts in relation to the NID group (Figures 1A and 1B). Statistical analysis showed significant differences between AD compared to the NID group and LP compared to the NID group with respect to proerythroblast count (p ≤ 0.05), and between groups AD and NID with respect to polychromatic erythroblast count (p = 0.019). Interestingly, all clinical groups, together with the LP and HP groups, showed decreases in the orthochromatic erythroblast count in comparison with the NID group (p ≤ 0.05). Pathological alterations such as erythrophagocytosis and erythroid dysplasia were not detected in the microscopic evaluation, but basophilic erythroblasts in the mitotic division stage were observed in some dogs in each of the clinical groups (data not shown). Figure 1C illustrates erythroblast cellularity in bone marrow, and shows proerythroblasts as well as basophilic, polychromatic and orthochromatic erythroblasts.

### Both clinical status and bone marrow parasite density decodes several alterations in the leucopoietic series

**Figure 2:**
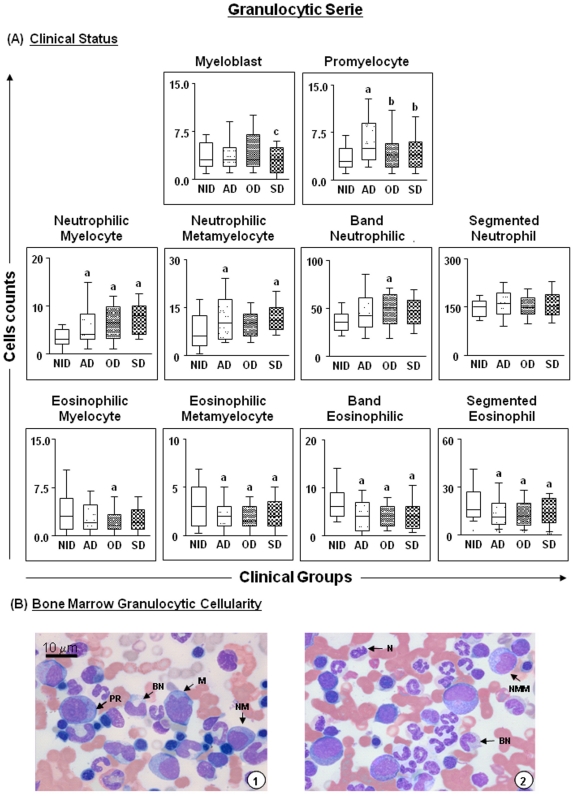
Profiles of the granulocytic cell series in dogs naturally infected with *Leishmania chagasi* categorised according to clinical status. Panel **A** - animals categorised according to their clinical status into asymptomatic (AD = white boxes with small points), oligosymptomatic (OD = white boxes with large points), symptomatic (SD = white boxes with squares) or non-infected (NID = white boxes) groups. Absolute cell counts are presented in a box-plot format, the median, maximum and minimum values represents the interquartile range. Significant differences (p<0.05) with respect to the NID, AD and OD groups are indicated by the letters ‘a’ and ‘b’ and ‘c’ respectively. Panel **B** - cellularity of the granulocytic series observed in the bone marrow of dogs assessed in this study. Slides were stained with Giemsa; bar  = 10 µm. M indicates myeloblast; PR, promyelocyte; NM, neutrophilic myelocyte; NMM, neutrophilic metamyelocyte; BN band neutrophil; N, neutrophil; EM, eosinophilic myelocyte; E, eosinophil.

**Figure 3:**
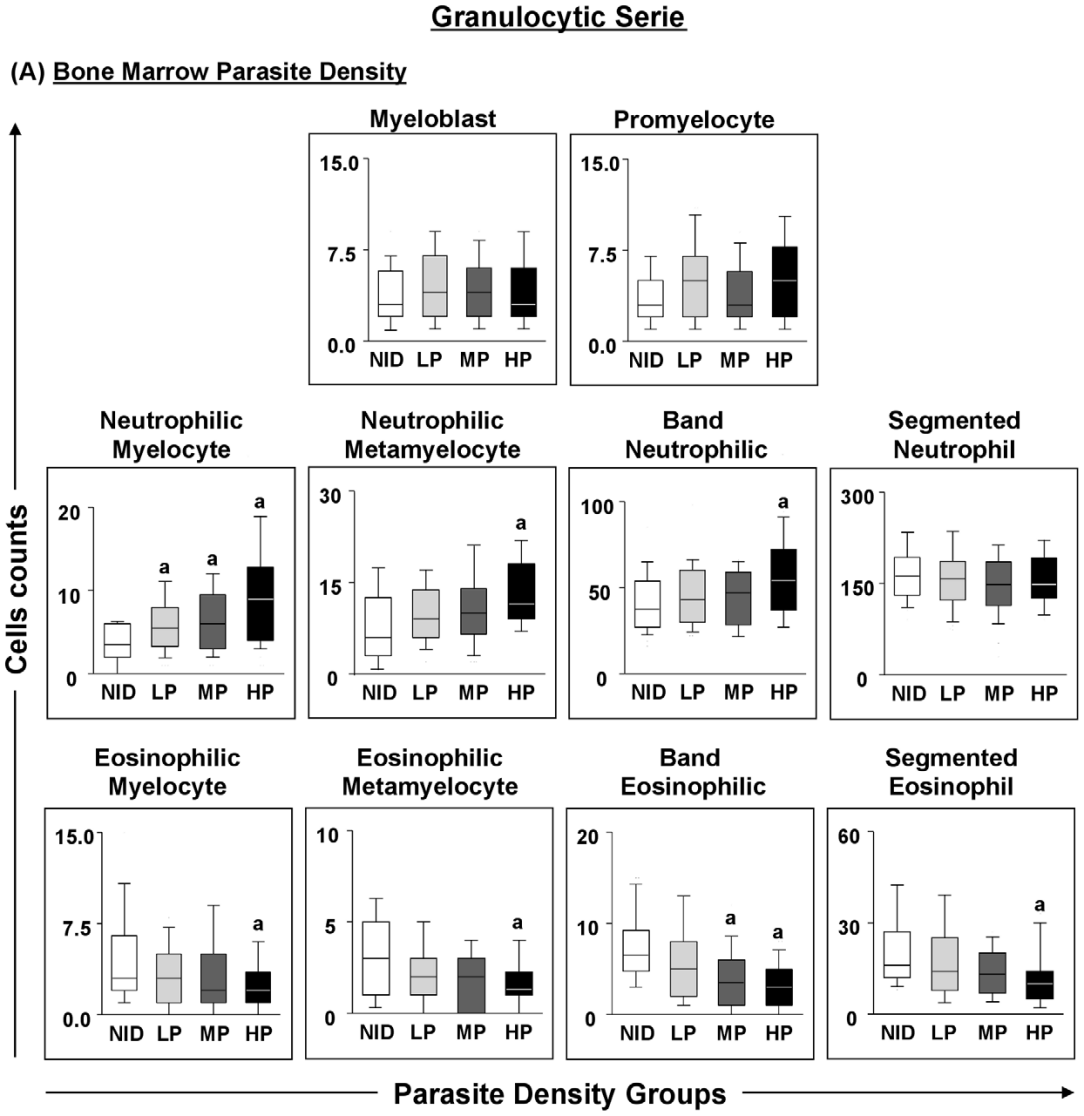
Profiles of the granulocytic cell series in dogs naturally infected with *Leishmania chagasi categorised according to parasite density*. Panel **A** - animals categorised according to bone marrow parasite density into low (LP = gray boxes), medium (MP =  dark gray boxes) and high (HP = black boxes) parasite density and non-infected (NID = white boxes) groups. Absolute cell counts are presented in a box-plot format, the median, maximum and minimum values represents the interquartile range. Significant differences (p<0.05) with respect to the NID group are indicated by the letter ‘a’.

Various alterations in leucopoiesis were observed in dogs infected with *Leishmania.* In the granulocytic series, there was a significant (p = 0.0246) reduction in myeloblasts in the SD group compared with the OD group, whereas the promyelocyte count in the AD group was increased in comparison with all other clinical groups and with the NID group (p = 0.0011) (Figure 2A). With respect to neutrophil precursors, there were increases in neutrophilic myelocytes in dogs included in all clinical groups and presenting different parasite densities in comparison with non-infected animals (p ≤ 0.05) (Figures 2A and 3A). Additionally, in comparison with the NID group, neutrophilic metamyelocytes were increased significantly (p ≤ 0.05) in the AD, SD and HP groups, whilst band neutrophils were increased significantly (p ≤ 0.05) only in the OD and HP groups.

In the eosinophil precursor series, significant (p≤0.05) decreases were observed in eosinophilic metamyelocytes, band eosinophils and segmented eosinophils in different groups of infected dogs compared with the NID group (Figure 2A). Additionally, in comparison with the NID group, eosinophilic myelocytes, metamyelocytes and segmented eosinophils were significantly (p≤0.05) reduced in the HP group, whilst band eosinophils were significantly (p<0.0001) decreased in the MP and HP groups (Figure 3A). Figure 2C illustrates granulocyte cellularity in bone marrow, and shows myeloblasts, promyelocytes, neutrophilic myelocytes and metamyelocytes, band neutrophils, neutrophils, eosinophilic myelocytes and eosinophils.

**Figure 4:**
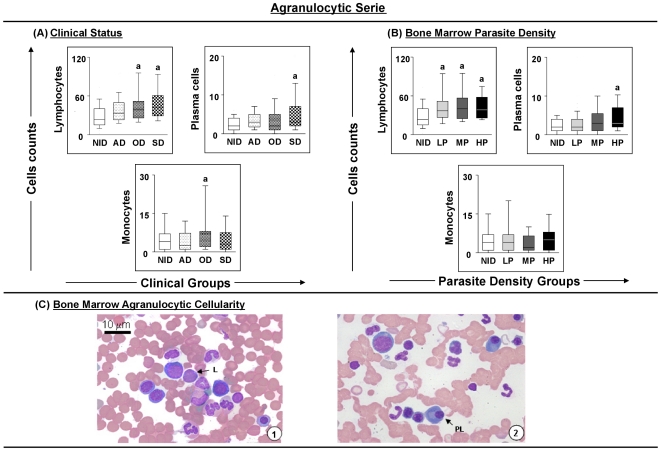
Profiles of the agranulocytic cell series in dogs naturally infected with *Leishmania chagasi*. Panel **A** - animals categorised according to their clinical status into asymptomatic (AD = white boxes with small points), oligosymptomatic (OD = white boxes with large points), symptomatic (SD = white boxes with squares) or non-infected (NID = white boxes) groups. Panel **B** - animals categorised according to bone marrow parasite density into low (LP = gray boxes), medium (MP = dark gray boxes) and high (HP = black boxes) parasite density and non-infected (NID = white boxes) groups. Absolute cell counts are presented in a box-plot format, the median, maximum and minimum values represents the interquartile range. Significant differences (p<0.05) with respect to the NID group are indicated by the letter ‘a’. Panel **C** - cellularity of the agranulocytic series observed in the bone marrow of dogs assessed in this study. Slides were stained with Giemsa; bar  = 10 µm. L indicates lymphocyte; PC, plasma cell; M, monocyte.

With respect to agranulocytic cells, significant (p = 0.0012) increases in the populations of lymphocytes were observed in the OD and SD groups, and in all parasite densities groups, in comparison with the NID group (Figures 4A and 4B). Similar results were obtained for plasma cells, which showed a clear tendency towards a gradual increase according to the severity of the infection (Figure 4A). Additionally, the monocyte count increased significantly (p = 0.0442) in the OD group compared with the AD group (Figure 4A). Figure 4C illustrates agranulocyte cellularity in bone marrow, and shows lymphocytes, plasma cells and monocytes.

### Parasite density in bone marrow is an efficient biomarker of disease prognosis and decodes clinical status during ongoing CVL

Distinct patterns of bone marrow parasite density were observed in dogs infected with *L. chagasi*. Parasitological assays revealed a significantly (p = 0.0249) higher LDU index in the SD group compared with the AD group.

### Both clinical status and parasite density modifies the maturation index ratios

Maturation index ratios relating to bone marrow cellularity were calculated with the aim of evaluating bone marrow response in dogs naturally infected with *L. chagasi* and exhibiting different clinical statuses and patterns of bone marrow parasite density (Table 2). The myeloid: erythroid (M:E) ratio and the maturation indices related to leucopoiesis (I:M), granulopoiesis (I:Mg) and erythropoiesis (I:Me) presented significant increases (p ≤ 0.05) in both the clinical groups and the parasite density groups in comparison with the NID group. In contrast, the myeloid maturation index (MMI: calculated as the inverse of the I:Mg ratio) and the erythroid maturation index (EMI; calculated as the inverse of the I:Me ratio) exhibited significant (p≤0.05) decreases in all of the clinical and parasite density groups when compared with the NID group (Table 2).

**Table 2:** Maturation Index Assessment of bone marrow cellularity in dogs naturally infected by *L. chagasi* presenting different clinical statuses and parasite densities.

Maturation IndexRatio	Clinical groups			
	NID	AD	OD	SD
M:E	1.40±0.60	2.20±1.30 a	2.10±1.30 a	2.00±1.10 a
I:M	0.06±0.02	0.09±0.06 a	0.08±0.04 a	0.08±0.03 a
I:Mg	0.05±0.03	0.08±0.06 a	0.08±0.04 a	0.07±0.03 a
MMI (1/I:Mg)	22.40±12.10	16.90±10.20 a	16.30±8.50 a	16.30±7.30 a
I:Me	0.20±0.08	0.30±0.10 a	0.30±0.10 a	0.30±0.10 a
EMI (1/I:Me)	4.90±2.10	3.50±1.50 a	3.60±1.30 a	3.70±1.50 a

	Parasite density groups			
	NID	LP	MP	HP
M:E	1.40±0.60	2.20±1.40 a	2.00±0.90 a	2.20±1.30 a
I:M	0.06±0.02	0.08±0.04 a	0.08±0.04^a^	1.00±0.06 a
I:Mg	0.05±0.03	0.08±0.04 a	0.08±0.04 a	0.09±0.05^a^
MMI (1/I:Mg)	23.00±11.90	16.00±6.40 a	14.90±9.30 a	15.00±9.60 a
I:Me	0.20±0.08	0.30±0.10 a	0.30±0.10 a	0.30±0.10 a
EMI (1/I:Me)	4.90±2.10	3.60±1.60 a	3.60±1.40 a	3.60±1.30 a

a Values statistically significantly different (p<0.05) from those of the NID group. NID indicates non-infected dogs; AD, asymptomatic dogs; OD, oligosymptomatic dogs; SD, symptomatic dogs; LP, low parasitism; MP, medium parasitism; HP, high parasitism; M:E, myeloid:erythroid; I:M, immature:mature; I:Mg, immature:mature (granulopoiesis); I:Me, immature:mature (erythropoiesis); MMI, myeloid maturation index; and EMI, erythroid maturation index.

## Discussion

Modifications in haematopoiesis are commonly associated with infection by viral, bacterial and protozoan pathogens. Thus, whilst haematopoiesis is suppressed in experimental infection with *Murine cytomegalovirus* and *Salmonella typhimurium*, it is increased in experimental murine malaria, schistosomiasis and leishmaniasis [23], [24]. However, very few studies have evaluated the impact of clinical status and distinct parasite density on haematopoietic activity during CVL.

The decreases in bone marrow proerythroblast and polychromatic erythroblast counts in asymptomatic infected dogs in comparison with non-infected animals, as demonstrated in the present study, corroborate the alterations in peripheral blood previously described as normocytic/normochromic anaemia [5], [25]. Additionally, we have demonstrated here for the first time that dogs infected with *L. chagasi* exhibit a decrease in the number of orthochromatic erythroblast cells characterising erythroid hypoplasia in CVL. Such a differential count indicates erythropoiesis with normal morphology and maturation sequence, as previous suggested Tryphonas *et al*. [26]. The observed increase in the I:Me ratio in both the clinical status and the parasite density groups revealed a greater proliferation of blast cells during severe CVL, whilst the EMI ratio was reduced by virtue of the hypoplasia present during the disease.

Several studies have reported myeloid hyperplasia in CVL [17], [26]. In the present study, myeloid hyperplasia was observed in the granulocytic series, but mainly in myeloblasts in the OD group and in promyelocytes in the AD group. Additionally, increases in neutrophilic myelocytes were established in all infected dogs, and higher neutrophilic metamyelocyte counts were found in the AD and SD groups. This phenomenon may be related to the proliferation of cellular precursors to provide the cell elements (neutrophils, eosinophils and basophils) required to meet the high demand of granulocytic cells. In this context, Cotterell *et al*. [24] showed that infection with *L. donovani* induced an increase in myelopoiesis (granulocytes and monocytes) with the mobilisation of the progenitors cells to the peripheral blood being associated with higher levels of granulocyte macrophage colony stimulating factor (GM-CSF) and colony forming units (GM-CFU). In fact, according to the clinical evolution of the disease, proliferation of neutrophilic cells can be explained as an attempt by the organism to control bone marrow parasite density. Thus, neutrophils could play a key role in controlling development of the initial *Leishmania* infection by destroying promastigotes forms through the activation of oxidative mechanisms.

An augmented M:E ratio is associated with myeloid hyperplasia resulting from increases in granulocyte cell lines, whilst a reduced M:E ratio is linked to erythroid hyperplasia resulting from increases in the erythropoietic series. In the present study, the observed increase in the M:E ratio indicated myeloid hyperplasia that was probably related to the increase of neutrophilic precursor cells as revealed by the rise in the I:Mg ratio in the clinical status and parasite density groups. The finding of an increased M:E ratio is in agreement with an earlier report [20]. As expected, the opposite result was found for MMI, which showed a decrease in all infected dogs indicating a defect in maturation in the neutrophilic cell series during CVL.

The eosinophils and their precursors were not responsible for the observed increases in the M:E and I:M ratios since eosinophilic hypoplasia was established in different clinical forms, suggesting a dysfunction of the bone marrow as indicated by the profiles of inflammatory cytokines in the different clinical phases of infection. Interestingly, some authors [5], [26] have described eosinopenia in the peripheral blood of symptomatic dogs during CVL, although Amusategui *et al*. [27] reported that eosinophil counts were higher in dogs that presented cutaneous signs associated with allergenic responses. It may be that the bone marrow eosinopenia observed in CVL is related to the preferential migration of eosinophils to the tissues. In this context, a microbicidal capability of eosinophils against *Leishmania donovani* and *Leishmania major* parasites has been demonstrated [28], [29]. It is important to note that interleukin 5 (IL-5) stimulates eosinophil production in the bone marrow by acting on colony forming unit-eosinophil (CFU-Eo) [30], and in CVL this cytokine has been associated with type 2 immune response (Th2). However, more studies are necessary in order to determine the role of IL-5 in eosinophil proliferation in the bone marrow during CVL.

Bone marrow lymphocytosis in oligosymptomatic and symptomatic dogs is most probably a compensatory response providing lymphocytes to the organs targeted by the infecting parasite. This condition may be associated with the lymphopenia observed in the peripheral blood of infected animals during advanced stages of CVL, which can be attributed to the immunosuppressive profile of VL [7], [31]. Although not significant, the reduced level of lymphocytes observed in asymptomatic dogs in the present study may be closely related to the lymphocytosis in peripheral blood in this group that had been previously reported [5]. This alteration appears to be closely associated with increases in circulating T lymphocytes CD4^+^ and CD8^+^ that are related to the maintenance of clinical morbidity in asymptomatic dogs and influence the parasite/host relationship [11].

The genesis of polyclonal B cell activation in CVL, which is responsible for the intense production of antibodies during the clinical course of the disease, may be associated with the plasmocytosis that is observed in symptomatic dogs. A recent report of a decrease in circulating B cells in SD dogs [11] could be explained by the selective migration of this cell population to the lymphoid organs or by loss of the CD21 B cell marker and, therefore, a decrease in B cells in lymphoid tissues [9]. Analysis of lymphoid organs from dogs naturally infected with *Leishmania* revealed enhanced areas of B cells, mainly plasma cells, associated with the increased production of anti-*Leishmania* antibodies as determined by serological tests [9], [14], [32]. These findings suggest that the migration of B cells from peripheral blood into lymphoid tissues might be occurring during active CVL, with activation and differentiation of these cells in the plasma cells and consequential polyclonal activity leading to a high production of anti-*Leishmania* antibodies.

An increase in circulating monocytes in the OD group in comparison with the NID group was observed in the present study. This finding could be related to a proliferation of these cells in an attempt to contain the intense bone marrow parasite density, leading to the destruction of the cell population and reflecting in peripheral blood monocytopenia mainly in symptomatic dogs [5]. In contrast, in animals infected with *L. donovani*, the migration of peripheral blood monocytes to hepatic granulomas [33], which is under the control of GM-CSF, generates leishmanicidal activity throughout macrophage activation [34]. Recently, we have reported hepatic alterations in CVL as an intense reaction of the Kupffer cells, capsule and portal inflammation, and the presence of intralobular granulomas [14]. These data may suggest that monocyte recruitment to lymphoid tissues occurs during active CVL, and that these cells might play an important role in immunological connection throughout antigen presentation and parasite clearance. However, we also observed high parasite densities in the lymphoid tissues including the spleen, liver, lymph node, bone marrow and skin [5], and this could be related to the inability of the macrophages to eliminate the parasites.

In order to evaluate the impact of parasite density on haematopoiesis in the present study, the animals were classified according to specific parasite density. The majority of changes that occurred in the bone marrow were observed in the HP group, demonstrating that the parasite load is associated with alterations in erythropoiesis and leucopoiesis including a decrease in orthochromatic erythroblasts, an increase in neutrophilic lineage proliferation, and reductions in eosinophilic lineage proliferation, lymphocytosis and plasmocytosis. These results complement previous studies that showed haemogram alterations as a decrease in the levels of red blood cells, haemoglobin and haematocrit, and the number of circulating white blood cells (eosinophils, lymphocytes and monocytes) in dogs with high bone marrow parasite densities [11].

In the present study, we have also assessed the impact of bone marrow parasite density (evaluated in the form of LDU indices) in different clinical groups of CVL. The results have shown that the bone marrow of symptomatic dogs exhibits higher parasite densities in comparison with asymptomatic and oligosymptomatic animals. In fact, several studies have reported reasonable correlations between spleen and lymph node parasite densities and clinical forms of CVL [4], [35]. In an earlier study we have demonstrated that bone marrow parasite density is the most reliable parasitological marker by which to decode the clinical status of CVL, since this organ provided a better correlation between parasite density and clinical form compared with the spleen, lymph node, skin or liver [5].

Taken together, the data presented herein highlight the bone marrow as an important organ in CVL and suggest an additional diagnostic tool by which to study the correlation of parasite density with erythropoiesis and leucopoiesis. Additionally, it is concluded that the evaluation of the bone marrow provides a useful method by which to elaborate a prognosis for CVL as well as for the diagnosis of cases involving a strong suspicion of CVL that could not be confirmed by serologic tests.
